# Determinants of patient-reported satisfaction with treatment outcome 6 months after rhinoplasty

**DOI:** 10.1016/j.jpra.2025.09.026

**Published:** 2025-09-25

**Authors:** Kim_Phi Luong, Stefan Hummelink, Bart Stubenitsky, Harm P. Slijper, Dietmar J.O. Ulrich

**Affiliations:** a Department of Plastic, Reconstructive and Hand Surgery, Radboud University Medical Center, Geert Grooteplein Zuid 106525 GA, Nijmegen, The Netherlands; b Research Center, Velthuis Kliniek, Flight Forum 1305657 DD, Eindhoven, The Netherlands; c Dr_BartClinic, Valeriusstraat 551071 MD, Amsterdam, The Netherlands; d Department of Plastic and Reconstructive Surgery, Erasmus MC, University Medical Center Rotterdam, Dr. Molewaterplein 40, 3015 GD, Rotterdam, The Netherlands

**Keywords:** Rhinoplasty, Patient satisfaction, Quality of life, Cosmetic, Cohort studies, Surveys and questionnaires

## Abstract

**Background:**

Rhinoplasty is a complicated facial surgery which aims to improve the appearance of the nose, resulting in the enhancement of satisfaction with appearance and quality of life. Therefore, it is crucial to understand which factors may influence satisfaction with treatment outcome.

**Methods:**

A cohort study was conducted in 215 patients undergoing rhinoplasty. Multivariable linear regression analysis was used to assess the influence of patient characteristics, baseline nasal characteristics, baseline patient-reported outcomes, and postoperative clinician-reported outcomes on satisfaction with treatment outcome 6 months post-rhinoplasty.

**Results:**

The final model included all preoperative and postoperative characteristics and the following variables were independent determinants that contributed to a higher outcome satisfaction: Patients with a dorsal hump preoperatively (β = 0.32; <0.001), over or under projection preoperatively (β = 0.13; <0.05) and a higher clinician-reported appraisal of the nose appearance (VAS-scale) postoperatively (β = 0.18; <0.01). A bulbous or boxy tip preoperatively (β = −0.13; <0.01) was associated with worse satisfaction with treatment outcome. According to the final model, 23 % of the variance in satisfaction with treatment outcome at 6 months after rhinoplasty could be explained.

**Conclusion:**

Baseline nasal characteristics and postoperative clinician-reported outcomes were associated with patient-reported satisfaction with treatment outcomes after rhinoplasty while patient characteristics and baseline patient-reported outcomes were not associated. Management of patient expectations regarding outcomes and the influence of baseline nasal characteristics could aid in improving patient-reported satisfaction with treatment outcome.

## Introduction

In recent years, there has been a growing trend in the use of patient-reported outcome measures (PROMs) in plastic surgery to evaluate patient satisfaction and quality of life (QoL).^1^^,^^2^ These instruments are procedure or anatomical region-specific and designed to be completed by the patient alone, providing valuable insights into the patient’s perspective. The use of PROMs has been implemented in clinical practice as routine outcome measurements by a growing number of centers. It could be utilized to investigate predictors that contribute to optimal outcomes and, moreover, to inform patients more precise resulting in improved communication and treatment guidance.^3^, ^4^, ^5^

Rhinoplasty is one of the most commonly performed aesthetic procedures worldwide and is considered to be a complex procedure in the field of aesthetic plastic surgery.^1^^,^^6^, ^7^, ^8^ Studies evaluated patient benefits after rhinoplasty and have sought to identify patient characteristics that are associated with the outcome of satisfaction or QoL following rhinoplasty.^9^, ^10^, ^11^ These include patient traits such as the acronym SIMON (single, immature, male, overly expectant, and narcissistic), as well as various age groups and a positive history of cosmetic surgery. However, it remains unclear how these variables should be interpreted in daily clinical practice, and to what degree they influence the variance in patient-reported satisfaction and QoL. Furthermore, it is uncertain if other characteristics are of interest.

The support of facial analysis with 2D images or 3D models during preoperative consultation may be of value due to expectation management but the specific role of nasal characteristics remains imprecise.^12^^,^^13^ Various studies have investigated which postoperative nasal characteristics are more common in negative outcomes but studies exploring preoperative nasal characteristics and their contribution on PROMs are scarce.^14^, ^15^, ^16^

Given these considerations, it would be beneficial to assist clinicians during the consultation by providing them with a clear understanding of the factors that influence patient satisfaction and QoL following rhinoplasty. This will allow them to communicate more effectively with their patients and provide them with more precise information about the potential outcome of the procedure.^17^^,^^18^

Therefore, this study aimed to assess which patient characteristics, nasal characteristics, clinician-reported post-surgical outcomes, and baseline PROMs are associated with patient satisfaction with the treatment outcome of the nose 6 months after primary rhinoplasty in order to enhance preoperative consultation.

## Methods

### Study design and setting

A multicenter observational cohort study was performed at four practice sites of the Velthuis Clinics, The Netherlands. All patients who underwent a primary rhinoplasty at Velthuis Clinic between December 2016 and December 2021 were invited to complete several online questionnaires. This was part of a routine outcome measurement system and was reported following the *Reporting of studies Conducted using Observational Routinely collected Data* (RECORD) guidelines.^19^ A similar system used for assessing outcomes regarding hand and wrist disorders is previously described by Selles et al.^3^ For data managing, GemsTracker, a secure web-based application for distributing questionnaires, was used.^20^ Patients who approved to participate received e-questionnaires after their first consultation and 6 months postoperatively. If the questionnaires were not completed, a reminder was sent for each round with a maximum of three reminders. This study was approved by the local Medical Ethical Review Committee (2020–6680), following the guidelines of the Declaration of Helsinki. Informed consent was obtained from all patients for an anonymized analysis of their data.

### Participants

All patients who underwent a primary open or closed preservation rhinoplasty between December 2016 and December 2021 were invited to complete multiple online questionnaires. Participants were excluded if they had another facial procedure simultaneously or within 6 months postoperatively or if they failed to complete all questionnaires.

Patient characteristics were obtained from the electronic database of the Velthuis Clinic and included gender, age, BMI, smoking status (yes/no), and cosmetic surgery in the past (yes/no). Patients who quit smoking less than 6 weeks before the procedure were categorized as smokers due to lingering negative effects on wound healing.^21^

### Nasal characteristics

A total of 6-view standardized head photos (frontal, oblique, and lateral views from both sides and base view) were taken at the first consultation and approximately 6 months postoperatively. The preoperative and postoperative photos were scored using a standardized questionnaire that collected data on nasal characteristics including dorsal lines (asymmetrical, wide, narrow, or ideal), dorsal hump (yes/no), radix-tip/stomion-menton (RT/SM) ratio, tip projection (over projection, >60 %; normal projection, 50–60 %; under projection, <50 %), tip shape (bulbous, boxy or normal), if the alar base width was greater compared to the intercanthal distance (yes/no) and a Visual Analogue Scale (VAS) rating the appearance of the nose (range 0–10, higher score indicating a greater cosmetic outcome). All photos were scored by a junior scientist (K.P.L) after being trained by two plastic surgeons (D.J.O.U. and V.P.).

### Utrecht questionnaire for outcome assessment in aesthetic rhinoplasty

The *Utrecht Questionnaire for Outcome Assessment in Aesthetic Rhinoplasty* (OAR), also known as the *Utrecht Questionnaire* (UQ), is a PROM to evaluate body image and QoL in relation to nasal appearance.^22^ The first part consists of a VAS rating the appearance of the nose from 0 (very ugly) to 10 (very nice). A higher score indicates a better outcome. The second part of the questionnaire consists of five questions scored on a 5-point Likert scale. A total sum score can be calculated to a range from 5 to 25, with a lower score indicating a better outcome. This questionnaire was completed preoperatively.

### FACE-Q aesthetics

The *FACE-Q Aesthetic* is a PROM used to evaluate surgical or minimally invasive aesthetic facial treatment.^23^^,^^24^ Satisfaction with outcome from the QoL domain was used as the primary outcome. This outcome measures satisfaction with the result of the facial procedure (e.g., statements such as *result being great*) and comprises six items scored on a 4-point Likert scale. The sum score is Rasch-transformed to a 0–100 scale, with higher scores indicating greater satisfaction. This scale was completed 6 months post-rhinoplasty considering the consistent outcomes of preservation rhinoplasty and a reduction of 95 % postoperative swelling.^25^ Moreover, this specific follow-up has been endorsed following a systematic review.^1^

### Statistical analysis

A complete case analysis was performed with participants who completed both questionnaires and had no missing photo documentation.

A four-step multivariable hierarchical linear regression analysis assessed the contribution of variable sets to satisfaction with treatment outcome at 6 months postoperatively. In the first step of the model, baseline patient characteristics including gender, age, BMI, smoking status, and previous cosmetic surgery were added. In the second step, baseline nasal characteristics were entered into the model. In the third step, the clinician-reported VAS regarding appearance postoperatively (range 0–10) was admitted to the model. In the fourth and last step, the baseline outcomes of the OAR including the VAS and sum score were included in the final model. Reports about PROMs and their association with satisfaction are scarce, and are therefore added as the last step while patient characteristics, the association between nasal topography and clinician-reported VAS have been investigated for various times.^9^^,^^14^^,^^26^

Regression results included unstandardized (B) and standardized (β) coefficients with 95 % CIs. B indicates the change in satisfaction per unit increase in an independent variable, holding others constant. β reflects the relative strength of each variable's association, allowing comparison across different scales. For each step, the explained variance (R^2^), the explained variance adjusted for the number of variables in the model (adjusted R^2^), and the significance of F-change are reported. Multicollinearity between variables is examined using the variance inflation factor (VIF), interpreted following Gareth’s guideliness.^27^

Considering data were collected via e-questionnaires and participation was not obligated, missing data were expected. Thus, a non-responder analysis was performed to determine if complete cases (completers) were different based on patient characteristics and baseline PROM scores from patients who only filled in the baseline questionnaire (non-completers). For this analysis, T-tests and Mann-Whitney-Wilcoxon tests were used for normal and non-normal continuous distributed data, respectively. Chi-square statistics for categorical data. Effect sizes were calculated to report the substantive significance, interpreted using Cohen’s criteria (0.2, small; 0.5, medium; 0.8, large) for numeric variables and using Cliff’s delta criteria (0.147, small; 0.33, medium; 0.474, large) for categorical variables.^28^, ^29^, ^30^

Analyses were conducted in R 3.6.3 (R Foundation for Statistical Computing, Vienna, Austria). A *p*-value of < 0.05 was considered statistically significant. To account for the issue of multiple comparisons, a Bonferroni correction with a threshold of *p*-value < 0.004 (0.05/14 variables), was applied.^31^

## Results

Between December 2016 and December 2021, a total of 1795 patients underwent a rhinoplasty, of which 1260 underwent a primary procedure. In total, 1131 patients were excluded because they did not provide informed consent, underwent a previous nasal surgery, underwent another facial cosmetic treatment simultaneously or within the follow-up period, or had incomplete baseline questionnaires.

**Figure 1:**
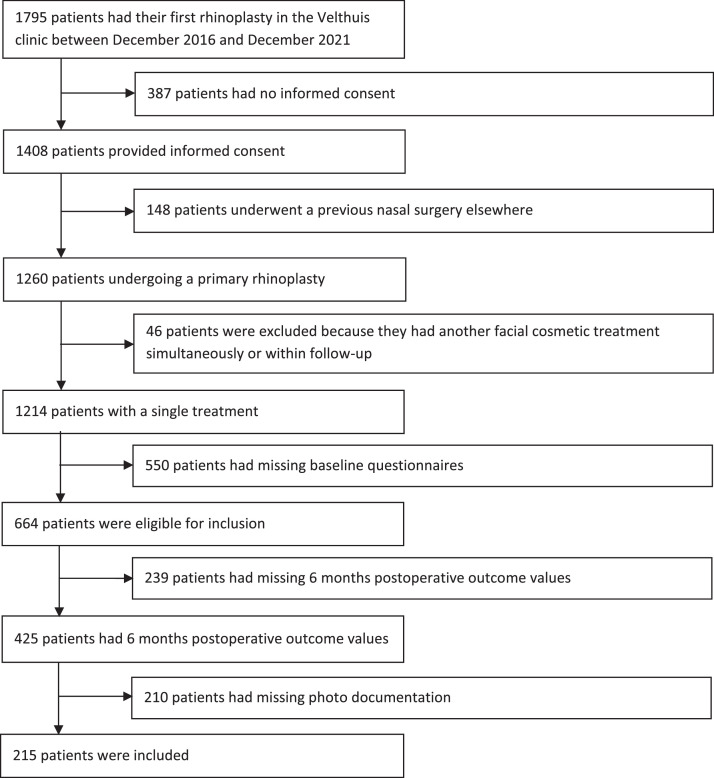
Flowchart of patient inclusion.

Of the 664 eligible patients, 425 patients (response rate 64 %) completed the questionnaires postoperatively. Among these patients, 210 patients had incomplete photo documentation, resulting in an enrollment of 215 patients in this study (Figure 1). These surgeries were operated by three plastic surgeons. The third author performed surgery on a total of 181 patients (84.2 %), while the other two plastic surgeons performed surgery on a total of 8 (3.7 %) and 26 (12.1 %) patients.

### Patient characteristics

The mean age was 26.8 ± 8.9 years ranging from 18 to 58. In total, 89.3 % (192 of 215) were female. Patient characteristics at baseline are shown in Table 1.

**Table 1:** Baseline patient characteristics (*N* = 215).

Variable	N (%)
Gender (female)	192 (89.3)
Age (years)^a^	26.79 ± 8.89
BMI (kg/m^2^)^a^	21.83 ± 3.60
Smoking status (yes)	25 (11.6)
Cosmetic surgery in the past	23 (10.7)

a mean ± SD.

### Hierarchic multivariable linear regression model

#### Model 1: Influence of patient characteristics (demographics)

From all the patient characteristics included in the model, no patient characteristics were significantly associated with satisfaction with treatment outcome. This model explained 4 % of the variance (Table 2, Model 1).

**Table 2:** Results from all steps of the multivariable linear regression model on the FACE-Q Aesthetics Satisfaction with Outcome six months after rhinoplasty. Unadjusted beta’s (B) with 95% confidence intervals (CI) and adjusted beta’s (β) are reported.

	Model 1		Model 2		Model 3		Model 4	
	B	β	B	β	B	β	B	β
Baseline patient characteristics								
Gender (male)	-2.15 [-12.34; 8.03]	-0.03	-3.97 [-13.54; 5.59]	-0.05	-3.99 [-13.40; 5.41]	-0.05	-3.94 [-13.41; 5.53]	-0.05
Age	-0.18 [-0.58; 0.22]	-0.07	-0.11 [-0.48; 0.27]	-0.04	-0.02 [-0.39; 0.35]	-0.01	0.01 [-0.37; 0.40]	0.01
BMI	0.28 [-0.63; 1.20]	0.04	0.68 [-0.19; 1.55]	0.10	0.62 [-0.23; 1.48]	0.10	0.65 [-0.21; 1.51]	0.10
Smoking status (No)	4.69 [-5.24; 14.63]	0.06	5.77 [-3.57; 15.11]	0.08	5.80 [-3.39; 15.00]	0.08	6.45 [-2.84; 15.74]	0.09
Cosmetic surgery in the past (No)	10.91 [-0.09; 21.92]	0.14	8.00 [-2.37; 18.36]	0.11	7.21 [-3.01; 17.42]	0.10	7.67 [-2.60; 17.95]	0.10
Baseline nasal characteristics								
Dorsal hump (Yes)			22.40 [13.65; 31.14]⁎†	0.33	21.80 [13.18; 30.42]⁎†	0.32	21.68 [13.04; 30.33]⁎†	0.32
Bulbous or boxy tip			-7.76 [-14.64; -0.88]⁎	-0.15	-6.62 [-13.44; 0.20]	-0.13	-6.86 [-13.71; -0.00]⁎	-0.13
Over or under projection of tip			6.80 [0.68; 12.91]⁎	0.14	6.88 [0.86; 12.90]⁎	0.14	6.48 [0.39; 12.57]⁎	0.13
Asymmetrical, wide or narrow dorsal lines			5.10 [-1.99; 12.19]	0.09	4.90 [-2.08; 11.88]	0.09	5.34 [-1.75; 12.42]	0.10
Alar base > intercanthal distance			3.34 [-4.90; 11.59]	0.05	2.54 [-5.59; 10.68]	0.04	2.61 [-5.55; 10.77]	0.04
No equal RT/SM ratio			-3.27 [-11.33; 4.79]	-0.05	-3.22 [-11.16; 4.71]	-0.05	-3.73 [-11.76; 4.29]	-0.06
Clinician-rated outcome measure								
Clinician-rated VAS appearance					3.79 [1.09; 6.49]⁎	0.18	3.84 [1.12; 6.57]⁎	0.18
Patient-reported outcome measures								
VAS appearance							-0.19 [-2.77; 2.40]	-0.01
OAR sum score							0.36 [-0.48; 1.19]	0.06
R^2^	0.04		0.20		0.23		0.23	
Adjusted R^2^	0.01		0.16		0.18		0.18	
Sig. F-Change	0.17		<0.001		0.01		0.57	

⁎ Statistically significant on a *p*-value < 0.05.

† Statistically significant on a Bonferroni-corrected *p*-value of < 0.004.

#### Model 2: Influence of patient characteristics plus baseline nasal characteristics

The second model increased the variance by 16 % by adding the baseline nasal characteristics. A preoperative dorsal hump (*B* = 22.40; β = 0.33) and over or under projection of the tip (*B* = 6.80; β = 0.14) were significantly related to greater satisfaction with treatment outcome (Table 2, Model 2). On the other hand, a preoperative bulbous or boxy tip (*B* = −7.76; β = −0.15) was significantly related to a worse satisfaction with treatment outcome. After Bonferroni's correction, only a preoperative dorsal hump remained statistically significant.

#### Model 3: Influence of patient characteristics, baseline nasal characteristics, and clinician-reported VAS regarding appearance

After adding the clinician-reported VAS regarding appearance, a preoperative bulbous or boxy tip was not associated with the treatment outcome. This implies that the clinician-reported VAS regarding appearance (*B* = 3.79; β = 0.18) has a shared variance with a preoperative bulbous or boxy tip and pushed this variable out of significance. The baseline nasal characteristics regarding a dorsal hump (*B* = 21.80; β = 0.32) and over or under projection of the tip (*B* = 6.88; β = 0.14) remained significantly related to the satisfaction with treatment outcome (Table 2, Model 3). An addition of 3 % of the variance in satisfaction with treatment outcome was explained by adding this step, resulting in a total of 23 % variance. After Bonferroni's correction, only a preoperative dorsal hump remained statistically significant.

#### Model 4: Influence of patient characteristics, baseline nasal characteristics, clinician-reported VAS regarding appearance, and baseline PROMs

After adding patient-reported baseline VAS regarding satisfaction with the appearance of the nose and the sum score of the QoL measured by the OAR, having a dorsal hump preoperatively (*B* = 21.68; β = 0.32) or having an over or under projection of the tip preoperatively (*B* = 6.48; β = 0.13) and a greater clinician-reported VAS regarding appearance (*B* = 3.84; β = 0.18) were significantly associated with greater satisfaction with the treatment outcome (Table 2, Model 4). Contrary, having a bulbous or boxy tip preoperatively (*B*= −6.86; β = −0.13) was significantly associated with worse satisfaction with the treatment outcome. From the four significant variables in the final model, the largest standardized regression coefficient (β = 0.32) was defined by having a dorsal hump preoperatively thus indicating the largest independent effect on satisfaction with treatment outcome of all variables analyzed in this study. Moreover, having a dorsal hump preoperatively was the only variable that survived the Bonferroni correction. Altogether, the final model explained 23 % of the variance in satisfaction with treatment outcome at 6 months postoperatively which was also observed in model 3. This indicated that baseline PROMs did not affected the variance and was not of added value in explaining the variance in treatment outcome postoperatively. There were no indications for multicollinearity in all models as the VIF ranged from 1.01 to 1.48.

### Non-responder analysis

The patient characteristics and baseline OAR scores between completers and non-completers are shown in Table 3. A significant difference was observed for age, cosmetic surgery in the past, and the VAS for appearance. Effect sizes were negligible to small and thus considered not clinically relevant.

**Table 3:** Non-responder analysis.

Patient characteristics N (%)	Non-responders *N* = 449	Responders *N* = 215	*P*-value	Effect size^d^
Gender (female)	398 (88.6)	192 (89.3)	0.903	0.007
Age (years)^a^^,^^e^	29.7 ± 10.8	26.8 ± 8.9	**0.001**	0.283
BMI (kg/m^2^)^a^	21.9 ± 2.7	21.8 ± 3.6	0.869	0.014
Smokers	64 (14.3)	25 (11.6)	0.419	0.026
Cosmetic procedure in the past	76 (16.9)	23 (10.7)	**0.046**	0.062
OAR scores^a^ at intake				
VAS appearance^b^	3.3 ± 1.4	3.1 ± 1.3	**0.018**	0.197
OAR sum score (range, 5–25)^c^	15.2 ± 4.5	15.5 ± 4.2	0.329	0.081

The non-responder analysis showed statistically significant differences in age, cosmetic procedure in the past, and VAS appearance. However, the effect size was negligible to small.

a Scores are mean ± standard deviation.

b VAS score: range, 0–10. Higher score indicates greater satisfaction with the appearance of the nose.

c OAR sum score: range, 5–25. Lower scores indicate higher quality of life and body image.

d Effect size: interpreted according to Cohen’s criteria; negligible, <0.2; small, 0.2; medium, 0.5; and large, 08 or Cliff’s delta criteria; negligible < 0.147; small, 0.147; medium, 0.33; large, 0.474.

e Statistically significant (*p* < 0.05).

## Discussion

The main aim of this study was to identify the factors associated with satisfaction with treatment outcome 6 months after rhinoplasty. Patient characteristics including gender, age, BMI, smoking status, and cosmetic surgery in the past were not found to be associated with satisfaction with treatment outcome after rhinoplasty. Also baseline PROM scores, such as VAS regarding satisfaction with the appearance of the nose and the sum score of the QoL measured by the OAR were surprisingly not associated. Factors that did influence outcome satisfaction were the occurrence of a dorsal hump preoperatively, an over or under projection of the tip preoperatively and a higher clinician-rated VAS appearance score postoperatively. Patients with a bulbous or boxy tip preoperatively were found to have a decreased level of satisfaction postoperatively. The largest proportion of variance in satisfaction with treatment outcome was attributed to the nasal characteristics set (16 %). Patient characteristics and clinician-reported VAS regarding appearance postoperatively only accounted for a small proportion (7 %) of this variance while PROMs proportion was regarded as negligible.

In the current study, the postoperative nasal characteristics were not taken into consideration since the aim of this study was ultimately to enhance preoperative consultation while most studies frequently focus on postoperative nasal characteristics. As a result, it is difficult to compare the outcomes to other studies. However, this study’s findings are in line with Khansa et al. who described that dissatisfaction was most often caused by under correction of the original deformity thus the presence of a change in dorsal hump to no residual hump may lead to greater satisfaction.^14^ This is also observed by Constantian et al. where satisfied patients had a dorsal hump preoperatively.^15^ Moreover, Vuyk et al. explained that patients with specific physical feature requests are better suited for rhinoplasty.^32^ This may explain why patients with a dorsal hump or under or over projection of the tip are excellent patients, and, therefore, obtained greater satisfaction postoperatively. However, surprisingly, patients with a bulbous or boxy tip were defined as less satisfied postoperatively. This may be due to the challenging aspect of reshaping the tip and therefore, resulting in an undesirable correction. In addition, Khansa et al. observed that one of the most common reasons for dissatisfaction was a bulbous tip postoperatively.^14^

The limitations of this study include the reduced sample size due to complete cases compared to the number of patients undergoing their primary rhinoplasty. Many patients were excluded due to incomplete questionnaires or missing photo documentation. Although participation in the outcome measurement system was voluntary, it was anticipated that some patients would fail to complete all questionnaires. It is plausible that a considerable number of dissatisfied patients or satisfied patients refuse to complete the questionnaires, leading to a potential distortion of the results towards favorable outcomes and improvement or unfavorable outcomes, respectively. Nonetheless, the non-responder analysis revealed no clinical differences with negligible to small effect sizes between the patients who completed all questionnaires with complete photo documentation compared to patients without photo documentation. Furthermore, the patient demographics in this study, including a mean age and majority of the female gender, are comparable to those found in other studies regarding rhinoplasty.^33^ Hence, it is likely that the results of the study are representative of the majority of patients undergoing rhinoplasty and relevant to most patients. At the same time, the predominance of a single surgeon ensures consistency in operative technique, which reduces variability and strengthens the internal validity of our findings.

Another limitation of this study is the large proportion of unexplained variance in satisfaction with treatment outcome, which may be due to factors such as patient experience, expectation, motivation, and psychosocial aspects.^9^^,^^10^^,^^34^, ^35^, ^36^ In addition, literature has suggested that patients seeking rhinoplasty due to cosmetic reasons have higher expectations, leading to more negative outcomes.^9^^,^^37^^,^^38^ Furthermore, psychologically unstable patients are recognized as being less ideal candidates. Thus, a significant proportion of the unexplained variance may be accounted for by these factors if they were assessed. In addition, given the modest sample size, incorporating multiple additional psychologically aspects or surgical variables would also have risked overfitting this model. Future research with larger cohorts and standardized operative reporting will be essential to clarify the impact of surgical technique on patient-reported outcomes.

Furthermore, the current utilized PROMs may be perceived as limited since functional aspects were not measured. However, it is important to emphasize that the included patients in this study primarily underwent the procedure for aesthetic reasons which reflects the setting of the clinic. Consequently, selecting the simplest and most patient-friendly questionnaires were deemed practical for daily clinical use. For future studies, it may also be of interest to add a functional PROM to understand the potential correlation between aesthetic and functional outcomes.^39^

Considerably more research is needed to determine which other factors could explain the large variance in satisfaction with treatment outcome 6 months postoperatively to elucidate the underlying mechanism behind patient-reported satisfaction with treatment outcome. Future research should focus on additional factors such as patient experience, expectations, and psychosocial aspects. Although bulbous or boxy tip and over or under projection did not survive the Bonferroni correction, it would be interesting to keep these variables in mind for future investigations since Bonferroni can be considered as overly conservative. Moreover, it would be interesting if all these variables combined could lead to predicting satisfaction with treatment outcome specific to the rhinoplasty population during the first consultation.

## Conclusion

Baseline nasal characteristics are strongly associated with satisfaction with treatment outcome 6 months after rhinoplasty. Patient characteristics, clinician-reported post-surgical outcomes, and baseline patient-reported outcomes are of limited value in explaining the satisfaction with treatment outcome. A significant proportion of the variance in satisfaction with treatment outcome remains unexplained, likely due to missing variables, such as patient experiences, expectations, and psychosocial aspects. To improve patient satisfaction, addressing the presence or absence of a dorsal hump, and potentially addressing the boxy or bulbous tip and the projection of the tip during preoperative consultation may aid in setting more realistic expectations. Optimizing patient counseling is essential to improve the patients’ expectations and ultimately enhance the outcome.

## Financial disclosure statement

None of the authors has a financial interest in any of the products, devices, or drugs mentioned in this manuscript.

## Funding

None.

## Statement of ethical approval

Approved by the local Medical Ethics Review Committee (2020–6680).

## Declaration of competing interest

None.

## References

[1] Wähmann M.S., Bulut O.C., Bran G.M., Veit J.A., Riedel F. (2018). Systematic review of quality-of-life measurement after aesthetic rhinoplasty. Aesthetic Plast Surg.

[2] Howldar S., Fida A., Allinjawi O., Zaqzoog F., Qurban G. (2018). Long-term cosmetic and functional outcomes of rhinoplasty: a cross sectional study of patients’ satisfaction. Saudi J Otorhinolaryngol Head Neck Surg.

[3] Selles R.W., Wouters R.M., Poelstra R. (2020). Routine health outcome measurement: development, design, and implementation of the hand and wrist cohort. Plast Reconstr Surg.

[4] AlHarethy S., SS Al-Angari, Syouri F., Islam T., Jang Y.J. (2017). Assessment of satisfaction based on age and gender in functional and aesthetic rhinoplasty. Eur Arch Otorhinolaryngol.

[5] Koybasi S., Bicer Y.O., Seyhan S., Kesgin S. (2018). Satisfaction in rhinoplasty: the possible impact of anxiety and functional outcome. Eur Arch Otorhinolaryngol.

[6] Barone M., Cogliandro A., Di Stefano N., Tambone V., Persichetti P. (2017). A systematic review of patient-reported outcome measures after rhinoplasty. European Archives of Oto-Rhino-Laryngology.

[7] Xiao H., Zhao Y., Liu L., Xiao M., Qiu W., Liu Y. (2019). Functional/aesthetic measures of patient satisfaction after rhinoplasty: a review. Aesthetic surgery journal.

[8] ISAPS International survey on aesthetic/cosmetic procedures performed in 2020. International Society of Aesthetic Plastic Surgery (ISAPS). https://www.isaps.org/media/evbbfapi/isaps-global-survey_2020.pdf (Accessed 16 october 2025)

[9] Herruer J.M., Prins J.B., van Heerbeek N., Verhage-Damen G., Ingels K. (2015). Negative predictors for satisfaction in patients seeking facial cosmetic surgery: a systematic review. Plast Reconstr Surg.

[10] Gorney M. (May 16, 2003). Presented at the Residents and Fellows Forum, Aesthetic Plastic Surgery Annual Meeting.

[11] Meyer L., Jacobsson S. (1986). The predictive validity of psychosocial factors for patients' acceptance of rhinoplasty. Ann Plast Surg.

[12] Lekakis G., Hens G., Claes P., Hellings P.W. (2019). Three-dimensional morphing and its added value in the rhinoplasty consult. Plast Reconstr Surg Glob Open.

[13] Persing S., Timberlake A., Madari S., Steinbacher D. (2018). Three-dimensional imaging in rhinoplasty: a comparison of the simulated versus actual result. Aesthetic Plast Surg.

[14] Khansa I., Khansa L., Pearson G.D. (2016). Patient satisfaction after rhinoplasty: a social media analysis. Aesthet Surg J.

[15] Constantian M.B., Lin C.P. (2014). Why some patients are unhappy: part 2. Relationship of nasal shape and trauma history to surgical success. Plast Reconstr Surg.

[16] Neaman K.C., Boettcher A.K., Do V.H. (2013). Cosmetic rhinoplasty: revision rates revisited. Aesthet Surg J.

[17] Velikova G., Booth L., Smith A.B. (2004). Measuring quality of life in routine oncology practice improves communication and patient well-being: a randomized controlled trial. J Clin Oncol.

[18] Steiger J.D. (2011). The rhinoplasty consult. Facial Plast Surg.

[19] Nicholls S.G., Quach P., von Elm E. (2015). The reporting of studies conducted using observational routinely-collected health data (record) statement: methods for arriving at consensus and developing reporting guidelines. PLoS One.

[20] Erasmuc MC, Equipe Zorgbedrijven GemsTracker (Accessed 16 October 2023).https://gemstracker.org.

[21] Sørensen L.T. (2012). Wound healing and infection in surgery. The clinical impact of smoking and smoking cessation: a systematic review and meta-analysis. Arch Surg.

[22] Lohuis P.J., Hakim S., Duivesteijn W., Knobbe A., Tasman A.J. (2013). Benefits of a short, practical questionnaire to measure subjective perception of nasal appearance after aesthetic rhinoplasty. Plast Reconstr Surg.

[23] Klassen A.F., Cano S.J., Scott A., Snell L., Pusic A.L. (2010). Measuring patient-reported outcomes in facial aesthetic patients: development of the FACE-Q. Facial Plast Surg.

[24] Klassen A.F., Cano S.J., Schwitzer J.A., Scott A.M., Pusic A.L. (2015). FACE-Q scales for health-related quality of life, early life impact, satisfaction with outcomes, and decision to have treatment: development and validation. Plast Reconstr Surg.

[25] Pavri S., Zhu V.Z., Steinbacher D.M. (2016). Postoperative edema resolution following rhinoplasty: a three-dimensional morphometric assessment. Plast Reconstr Surg.

[26] Rohrich R.J. (1999). Streamlining cosmetic surgery patient selection–just say no!. Plast Reconstr Surg.

[27] James G., Witten D., Hastie T., Tibshirani R. (2013). An Introduction to Statistical Learning: With Applications in R.

[28] Cohen J. (1992). A power primer. Psychol Bull.

[29] Romano J., Kromrey J. (2006).

[30] Cliff N. (1996).

[31] Knudby A., Ellsworth L., Bonferroni C.E. (1936). Teoria statistica delle classi e calcolo delle probabilita, Pubblicazioni del R Istituto Superiore di Scienze Economiche e Commerciali di Firenze, 8: 3− 62. Brenning, A., 2009. Benchmarking classifiers to optimally integrate terrain analysis and multispectral remote sensing in automatic. Environment.

[32] Vuyk H.D., Zijlker T.D. (1995). Psychosocial aspects of patient counseling and selection: a surgeon's perspective. Facial Plast Surg.

[33] Schwitzer J.A., Albino F.P., Mathis R.K., Scott A.M., Gamble L., Baker S.B. (2015). Assessing demographic differences in patient-perceived improvement in facial appearance and quality of life following rhinoplasty. Aesthet Surg J.

[34] Tsehaie J., van der Oest M.J.W., Poelstra R. (2019). Positive experience with treatment is associated with better surgical outcome in trapeziometacarpal osteoarthritis. J Hand Surg Eur.

[35] van der Oest M.J.W., Hoogendam L., Wouters R.M. (2021). Associations between positive treatment outcome expectations, illness understanding, and outcomes: a cohort study on non-operative treatment of first carpometacarpal osteoarthritis. Disabil Rehabil.

[36] Blackburn J., van der Oest M.J.W., Chen N.C. (2021). Are patient expectations and illness perception associated with patient-reported outcomes from surgical decompression in de quervain's tenosynovitis?. Clin Orthop Relat Res.

[37] Kandathil C.K., Patel P.N., Spataro E.A., Most S.P. (2021). Examining preoperative expectations and postoperative satisfaction in rhinoplasty patients: a single-center study. Facial Plast Surg Aesthet Med.

[38] Metin M., Avcu M. (2021). The effect on patient satisfaction of the postoperative nasal topographic, demographic, and functional results of open and closed septorhinoplasty techniques. J Craniofac Surg.

[39] Radulesco T., Penicaud M., Santini L., Thomassin J.M., Dessi P., Michel J. (2018). Outcomes of septorhinoplasty: a new approach comparing functional and aesthetic results. Int J Oral Maxillofac Surg.

